# Perceived discrimination, anxiety and mood disorders among university students during the COVID-19 era: evidence from a cross-sectional survey in a Ghanaian public university

**DOI:** 10.3389/fpsyg.2023.1274585

**Published:** 2024-01-12

**Authors:** Eugene K. M. Darteh, Jerry Paul K. Ninnoni, Joshua Okyere, Florie Darteh, Johannes John-Langba, Kwamena Sekyi Dickson

**Affiliations:** ^1^Department of Population and Health, University of Cape Coast, Cape Coast, Ghana; ^2^Department of Mental Health, University of Cape Coast, Cape Coast, Ghana; ^3^School of Nursing and Midwifery, College of Health Sciences, Kwame Nkrumah University of Science and Technology, Kumasi, Ghana; ^4^Department of Research and Advocacy, Challenging Heights, Winneba, Ghana; ^5^Institute of Education, University of Cape Coast, Cape Coast, Ghana; ^6^School of Applied Human Sciences, University of Kwazulu-Natal, Durban, South Africa

**Keywords:** discrimination, anxiety, mood disorders, mental health, social determinants

## Abstract

**Introduction:**

Before 2020 and the advent of the COVID-19 pandemic, mental disorders, including anxiety and mood disorders, were considered the leading causes of the global disease burden. There is evidence from multiple countries and social contexts that suggest the high risk of anxiety and mood disorders among students. Yet, there is a knowledge gap concerning understanding the association between the experience of discrimination and the risk of anxiety and mood disorders. We examined the association between the experience of discrimination and the risk of anxiety and mood disorders among university students.

**Methods:**

This study is a cross-sectional survey among university students in Ghana. A quota sampling technique was used to recruit 1,601 students. Data were collected using structured questionnaires. All data were analyzed using Stata. Binary logistic regression model was used to examine the significant association between the outcome variable and the explanatory variables.

**Results:**

The prevalence of anxiety disorder among the respondents was 67 per cent. Students who had experienced discrimination or had any member of their family experienced discrimination had higher odds (OR = 4.59, Cl = 2.64, 7.96) of anxiety and mood disorder compared to those who had not experienced any form of discrimination. Respondents aged 20–24 years had higher odds (OR = 1.47, Cl = 1.16, 1.85) of anxiety and mood disorder than those aged 15–19. Students with a high perceived risk of contracting COVID-19 had a higher odd (OR = 1.52, CI = 1.10, 2.10) compared to those with a low perceived risk.

**Conclusion:**

The findings underscore a need for university authorities to lay out clear initiatives that will reinforce and meet the mental health needs of university students during and after periods of crisis, such as returning from COVID-19 lockdown. There must be a conscious effort to advocate and raise students’ awareness of anxiety disorders. Also, it is imperative to create support groups within the university set up to address the mental health needs of all students. Younger students should be the primary focus of these interventions.

## Introduction

Before 2020, mental disorders, including generalized anxiety disorders (GAD) and mood disorders, were the leading causes of the global disease burden (Rominski et al., 2017; Santomauro et al., 2021). The global mental disorders burden has increased after the COVID-19 pandemic (Pan et al., 2021). For instance, Santomauro et al. (2021) estimated that globally, there was an additional 76.2 million anxiety disorders during the pandemic in 2020, indicating an anxiety disorder prevalence of 4,802 cases per 100,000 population. Estimates from the World Health Organization (WHO) shows that in 2016, GAD was among the leading contributors to DALY’s loss among women aged 15–29 in Africa (World Health Organization [WHO], 2016). In a related study in Ghana, the general population reported a 53.6% prevalence of GAD (Amu et al., 2021).

There is undeniable evidence from multiple countries and social contexts that suggest a high risk of GAD and mood disorders among students and adolescents (Bettmann et al., 2019; Kuringe et al., 2019; Ahulu et al., 2020; Charles et al., 2021; Faisal et al., 2022). This has been attributed to the effects culminating from academic demands (Radeef et al., 2014; Mofatteh, 2021) and financial stress (Usman and Banu, 2019; Jessop et al., 2020). Studies have also observed associations between GAD and academic performance (Liu et al., 2023), suicidal ideation (O’Neil Rodriguez and Kendall, 2014) and the risk of suicide significantly among students (Trindade Júnior et al., 2021). For example, Liu et al. (2023) revealed in their study that anxiety has heterogenous effects. That is, the more anxious students felt, the less likely they were to pursue goals focused on mastering new things. However, anxiety exerted positively on freshman students’ mastery goals (Liu et al., 2023).

Generalized anxiety disorders (GAD) and mood disorders are not limited to one stream of factors; instead, it is an interplay of various levels of systems that interact (Bronfenbrenner and Ceci, 1994; Ahulu et al., 2020). Hence, beyond the stressors, there are established determinants of anxiety and mood disorders among students during crisis situations such as the COVID-19 pandemic. These include gender differences in the risk of anxiety and mood disorders (Santomauro et al., 2021), knowledge of COVID (Quansah et al., 2022), and racial/ethnic differences (Charles et al., 2021).

Environmental factors such as having personally experienced or witnessed discrimination could potentially exacerbate the risk of anxiety and mood disorders (Stein et al., 2019; Cuevas et al., 2021). The association between experience of discrimination and risk of anxiety and mood disorders has not been investigated in any existing empirical studies in Ghana. Thus, suggesting an existing knowledge gap within the Ghanaian context. The present study seeks to bridge this knowledge gap by examining the association between discrimination and the risk of anxiety and mood disorders among university students in Ghana.

The study contributes novelty to the scientific community in many ways. First, it addresses a significant gap in existing knowledge by specifically examining the association between discrimination and anxiety/mood disorders among university students. This focused approach adds depth to our understanding of the psychosocial determinants of mental health. Moreover, the study’s focus on university students in Ghana contributes to a more diverse and representative understanding of mental health. Often, psychological research has been skewed toward Western and Asian contexts (Bunting et al., 2022; Cao and Liu, 2022; Cao, 2023), and this study helps bridge that gap by exploring mental health issues in a different cultural and sociopolitical context. Also, by investigating mental health outcomes in the aftermath of the pandemic, the study provides insights into the unique challenges faced by university students during this specific period.

## Literature review

Perceived discrimination, defined as the subjective awareness of unfair treatment based on one’s race, gender, ethnicity, or other social characteristics, has long been associated with negative mental health outcomes. Studies conducted prior to and during the pandemic consistently demonstrated a strong link between perceived discrimination and increased levels of anxiety and mood disorders among diverse populations. For example, Kogan et al. (2022) in their study observed that individuals who experienced more discrimination and microaggression were more likely to experience GAD than those who were not discriminated. The results of another study (Moody et al., 2023) indicates that individuals who experience major discrimination vicariously and encounter personal instances of everyday discrimination exhibit elevated levels of GAD. However, as stated already, these studies have been conducted in jurisdictions other than Ghana. Given the unique sociocultural context of university students in Ghana, it is imperative to understand the extent the which perceived discrimination predicts GAD and mood disorder. Therefore, this study tests the hypothesis that:

Perceived discrimination has a positive significant association with the risk of GAD and mood disorder among university students.

## Materials and methods

### Analytical cross-sectional survey

The study uses data from a survey of residential students at the University of Cape Coast. The University of Cape Coast is one of Ghana’s fifteen state-owned public universities. It has a population of around 24,000 students enrolled in various academic programs. More than 8,300 residential students are housed in the University’s eight designated residence halls and hostels. The University has a residential policy in which all freshmen are housed in traditional residence halls while continuing students choose their housing arrangements (Rominski et al., 2017). We argue that the result in this university is mostly likely to be similar in other public universities in Ghana since all have similar socio-cultural characteristics. Hence, the University of Cape Coast was randomly selected from the list of public universities in Ghana.

### Instrument

The questionnaires were developed, and pilot tested among students (*n* = 50) who studied at the Cape Coast Technical University in the same city. Some slight modifications were made to the survey based on these pilot tests. The survey was self-administered on tablet computers using Kobo Toolbox software. The instrument had four sections: (a) socio-demographic information, (b) fear of COVID-19, (c) Generalized Anxiety Disorder (GAD)-7 scale.

### Sampling

Based on the population of each hall of residence, a quota was allotted. A list of room numbers was prepared for each hall, and rooms included in the survey were chosen randomly using a random number generator. Research assistants addressed each room and explained the project to the first occupant they met. Students who consented to participate were given tablets with the survey on them. Respondents were encouraged to complete the survey privately to preserve privacy and confidentiality. It took roughly 20 min to complete each survey. A total of 1674 residential students were interviewed successfully.

### Ethical considerations

The University of Cape Coast’s Ethical Committee approved the survey protocol (UCCIRB/EXT/2021/15). Six field assistants were trained on the survey’s aims and were on hand to answer any respondents’ queries as they completed the survey. The field assistants could not see the participants’ responses, and no personal information was recorded.

### Measurement of variables

#### Outcome variable

The outcome variable generalized anxiety disorder (GAD) was derived from seven questions. Feeling nervous, anxious and on the edge in the past 2 weeks (not at all—0, several days—1, more than half the days—2, nearly every day—3); not being able to sleep or control worrying in the past 2 weeks (not at all—0, several days—1, more than half the days—2, nearly every day—3); worrying too much about different things in the past 2 weeks (not at all—0, several days—1, more than half the days—2, nearly every day—3); trouble relaxing in the past 2 weeks (not at all—0, several days—1, more than half the days—2, nearly every day—3); being so restless that it is hard to sit still in the past 2 weeks (not at all—0, several days—1, more than half the days—2, nearly every day—3); becoming easily annoyed or irritable in the past 2 weeks (not at all—0, several days—1, more than half the days—2, nearly every day—3); feeling afraid as if something awful might happen in the past 2 weeks (not at all—0, several days—1, more than half the days—2, nearly every day—3) (Rutter and Brown, 2017). The responses to the were recoded as (not at all was recoded as “not anxious” and several days, more than half the days, nearly every day were recoded as “anxious”). An index was generated for all the not at all and the other responses with scores ranging from 0 to 7. The score 0 was labeled as “not anxious” and 1 to 7 was labeled as “anxious.” A dummy variable was generated with “0” score being respondents who answered not anxious for all the 7 question and “1” if the respondents who answered “not anxious” for all the 7 questions. The Cronbach alpha and scale reliability coefficients was 0.7788. Which means that the GAD-scale is reliable.

#### Explanatory variables

These variables included age (15–19—1, 20–24—2, 25–29—3, 30+—4), sex of respondent (male—1, female—2), marital status (never married—1, married—2, separated—3, divorced—4, widowed—5), member of your family experienced any form of discrimination (no—1, yes—2), perceived risk of contracting COVID-19 (low—1, moderate—2, high—3).

### Analyses

All data were transferred from the Kobo Toolbox platform to Excel and imported into Stata. First, descriptive statistics were used to explore the data. Finally, a binary logistic regression model was used in multivariate analysis to examine the significant association between the outcome variable (generalized anxiety disorder) and the explanatory variables (background characteristics). A binary logistic regression model was utilized based on the outcome variable’s dichotomous nature. The results were presented in odds ratio at a 95% confidence interval (CI).

## Results

### Background characteristics

A total of 1,601 students were included in the study. Sixty-three percent of the respondents were males. Six in 10 were aged 20–24 years old and 98 percent were never married. One in 10 had either experienced or had a family member experiencing a form of discrimination and 55 percent perceived they had a low risk of contracting COVID-19 (see Table 1).

**TABLE 1:** Background characteristics.

Variable	Frequency (*n* = 1,601)	Percentage
**Age of respondent**		
15–19	512	32.0
20–24	1,014	63.3
25–29	67	4.2
30+	8	0.5
**Sex of respondent**		
Male	1,016	63.5
Female	577	36.0
Prefer not to say	8	0.5
**Marital status**		
Never married	1,570	98.1
Married	24	1.5
Separated/divorced	7	0.4
**Have you or any member of your family experienced any form of discrimination**		
No	1,435	89.6
Yes	166	10.4
**Perceived risk of contracting COVID-19**		
Low	886	55.3
Moderate	448	28.0
High	267	16.7
Total	1,601	100

### Generalized anxiety disorder

Twenty-one percent of respondents had been feeling nervous, anxious and on edge for several days. About 20 percent of had not been able to sleep or control worrying for several days, 30 percent had been worrying too much about different things for several days, 16 percent had trouble relaxing for several days, nearly 11 percent had been so restless that it is hard to sit still for several days, approximately 19 percent had been becoming easily annoyed or irritable for several days and 26 percent are feeling afraid that something awful might happen (see Table 2).

**TABLE 2:** Generalized anxiety disorder (GAD).

GAD variable	Frequency	Percentage
**Feeling nervous, anxious and on the edge**		
Not at all	1,173	73.3
Several days	340	21.2
More than half the days	52	3.3
Nearly every day	35	2.2
**Not being able to sleep or control worrying**		
Not at all	1,157	72.3
Several days	313	19.5
More than half the days	91	5.7
Nearly every day	40	2.5
**Worrying too much about different things**		
Not at all	861	53.8
Several days	488	30.5
More than half the days	151	9.4
Nearly every day	101	6.3
**Trouble relaxing**		
Not at all	1,189	74.3
Several days	260	16.2
More than half the days	85	5.3
Nearly every day	67	4.2
**Being so restless that it is hard to sit still**		
Not at all	1,290	80.6
Several days	173	10.8
More than half the days	78	4.9
Nearly every day	60	3.7
**Becoming easily annoyed or irritable**		
Not at all	1,128	70.4
Several days	302	18.9
More than half the days	96	6.0
Nearly every day	75	4.7
**Feeling afraid as if something awful might happen**		
Not at all	993	62.0
Several days	423	26.4
More than half the days	120	7.5
Nearly every day	65	4.1

### Background characteristics, proportion with generalized anxiety and chi square

The prevalence of generalized anxiety disorder among the respondents was 67 percent. Respondents aged 20–24 years (70.6%), females (69.0%), who were married (79.2%), who had either experienced or had a member of their family experienced any form of discrimination (91.0%) and those who perceived have a high risk of contracting COVID-19 (75.3%) (see Table 3).

**TABLE 3:** Background characteristics, proportion with generalized anxiety disorder and chi square.

Variable	Frequency (*n* = 1,601)	Proportion with generalized anxiety disorder	Chi square (*P*-value)
**Age of respondent**			*X*^2^ = 15.8 (0.001)
15–19	512	61.1	
20–24	1,014	70.6	
25–29	67	62.7	
30+	8	50.0	
**Sex of respondent**			*X*^2^ = 1.7 (0.436)
Male	1,016	66.0	
Female	577	69.0	
Prefer not to say	8	75.0	
**Marital status**			*X*^2^ = 2.7 (0.257)
Never married	1,570	66.9	
Married	24	79.2	
Separated/divorced	7	85.7	
**Have you or any member of your family experienced any form of discrimination**			*X*^2^ = 47.6 (0.000)
No	1,435	64.4	
Yes	166	91.0	
**Perceived risk of contracting COVID-19**			*X*^2^ = 42.5 (0.000)
Low	886	60.3	
Moderate	448	75.9	
High	267	75.3	
Total	1,601	67.5	

### Binary logistic regression

Experienced any form of discrimination, age of respondents, and perceived risk of contracting COVID-19 have significant associations with anxiety and mood disorder. Students who had experienced discrimination or had any member of their family experienced discrimination had a higher odd (AOR = 4.59, Cl = 2.64, 7.96) of anxiety and mood disorder compared to those who had not experienced any form of discrimination. Respondent aged 20–24 years had a higher odd (AOR = 1.47, Cl = 1.16, 1.85) of anxiety and mood disorder compared to those age 15–19 years. Students with high perceived risk of contracting COVID-19 had a higher odd (AOR = 1.52, CI = 1.10, 2.10) compared to those with low perceived risk of contracting COVID-19 (see Table 4).

**TABLE 4:** Binary logistic regression.

Variable	Model I Odds ratio (confidence interval)	Model II Adjusted odds ratio (confidence interval)
**Have you or any member of your family experienced any form of discrimination**		
No	Ref	Ref
Yes	5.57***(3.24, 9.57)	4.59***(2.64, 7.96)
**Age of respondent**		
15–19		Ref
20–24		1.47**(1.16, 1.85)
25–29		0.88 (0.50, 1.56)
30+		0.48 (0.10, 2.31)
**Sex of respondent**		
Male		Ref
Female		1.18 (0.94, 1.48)
Prefer not to say		1.76 (0.32, 0.96)
**Marital status**		
Never married		Ref
Married		2.33 (0.80, 6.82)
Separated/divorced		3.66 (0.35, 38.33)
**Perceived risk of contracting COVID-19**		
Low		Ref
Moderate		1.88***(1.45, 2.44)
High		1.52**(1.10, 2.10)
**Model fit**		
LR Chi2 (10)	103.18	
Prov > chi2	0.000	
Pseudo R2	0.0509	
Log likelihood	−962.07245	

Ref = reference category **p < 0.01; ***p < 0.001.

## Discussion

The study examined discrimination, anxiety and mood disorders and the fear of COVID-19 among university students. Overall, there was a high prevalence of GAD among the studied university students. Our observed prevalence of 67% is higher compared to a similar study by Amu et al. (2021), who reported a 53.6% prevalence of GAD in the general population. The present study’s estimated prevalence of anxiety disorder is also higher than the 32% that was estimated in a study (Irby-Shasanmi and Erving, 2022) conducted among German students 20 months after the first COVID-19 restriction. Our findings suggests that university students in Ghana are particularly vulnerable to GAD. Thus, underscoring a need for university authorities to develop and implement practical initiatives to ease the anxieties of students especially during periods of crises such as the COVID-19 pandemic.

Consistent with previous literature (Sosoo et al., 2020; Irby-Shasanmi and Erving, 2022; Majumdar et al., 2022), we found a positive significant association between experiencing discrimination and GAD among university students. Specifically, individuals who have personally been discriminated or had witnessed a relative being discriminated were 4.59 times more likely to have GAD. A plausible explanation for this association is that discrimination exacerbates social exclusion and isolation (Brandt et al., 2022). This can result in feelings of hopelessness, loneliness and trauma which are known risk factors of GAD.

Students with moderate to high perceived risk of contracting COVID-19 had a significantly higher likelihood to develop GAD compared to those with lower perception of risk. The result aligns with the findings of a study conducted in Germany (Irby-Shasanmi and Erving, 2022) that found a 1.3 higher risk of GAD among students who were worried about contracting COVID-19. Our findings are also consistent with a related study from the United States (Son et al., 2020) that reported significantly higher odds of GAD among students who were worried about being infected or reinfected with COVID-19. Theoretically, the findings can be explained by the cognitive-behavioral model which describes the relationship between cognitions, emotions and behaviors in the onset and maintenance of psychological disorders (Hofmann, 2014). In the lens of the cognitive-behavioral model, individuals who perceive themselves to be at higher risk of contracting the virus may be more likely to engage in maladaptive cognitive processes such as catastrophic thinking, overestimating the likelihood and severity of negative outcomes, and underestimating their own ability to cope with the situation (Baartmans et al., 2022). These cognitive biases can lead to heightened levels of GAD.

It is indicative from the study that younger students were more likely to have GAD compared to older students. Similar pattern of association has been reported in Tee et al.’s (2020) study. It is possible to explain this observation from the perspective that unlike older students who are better at regulating their emotions, younger students are often lacking in this regard. Thus, explaining the high risk of anxiety disorders among this group.

## Strengths and limitations

The sample used is large enough to generalize the findings to entire population of university students. Nevertheless, the use of a cross-sectional design does not permit us to make any sort of causal extrapolations of our findings. Also, the use of a quantitative research approach means that we missed an opportunity to deeply explore and gain comprehensive insights into the anxiety disorder of university students. Since we relied on self-reported data, there is the possibility of recall bias and social desirability bias.

## Implications for policy and practice

While it is true that the immediate crisis has been largely mitigated worldwide, the profound and lasting impact of the pandemic on mental health continues to resonate. Our research, conducted during the COVID-19 era, not only captures a pivotal moment in time but also provides enduring insights for shaping educational practices in the aftermath of this global health crisis. In a post-pandemic landscape, where the mental wellbeing of students remains paramount, our study serves as a guidepost for institutions seeking to implement targeted interventions and support structures. Emphasizing this enduring relevance, our research not only contributes to the understanding of immediate challenges but also to the formulation of sustainable strategies that promote the long-term psychological health of university students.

Practically, it emphasizes a need for Ghana’s universities to establish or enhance mental health support services, providing accessible counseling and resources for students dealing with GAD and mood disorders. Furthermore, establishing peer support groups or mentorship programs within the university setting can create a sense of community and facilitate open discussions about mental health. These groups can be tailored to specific needs, such as those related to discrimination or pandemic-related stressors. It is imperative for Ghana’s universities to actively implement and enforce anti-discrimination policies. This involves creating an inclusive environment where all students feel respected and valued, contributing to a positive impact on mental health.

## Conclusion

In conclusion, there is a high prevalence of anxiety disorder among university students after the lifting of the first COVID-19 nationwide restrictions. Discrimination, perceived risk of contracting COVID-19, and age were the associated factors of anxiety disorders among the students. The findings underscore a need for university authorities to lay out clear initiatives that will reinforce and meet the mental health needs of university students during and after periods of crises, such as returning from COVID-19 lockdown. Practically, there must be a conscious effort to advocate and raise students’ awareness of anxiety disorders. Also, it is imperative to create support groups within the university set up to address the mental health needs of all students. Younger students should be the primary focus of these interventions.

## Data availability statement

The raw data supporting the conclusions of this article will be made available by the authors, without undue reservation.

## Ethics statement

The studies involving humans were approved by the University of Cape Coast’s Institutional Review Board. The studies were conducted in accordance with the local legislation and institutional requirements. The participants provided their written informed consent to participate in this study.

## Author contributions

ED: Conceptualization, Project administration, Supervision, Validation, Writing – original draft, Writing – review & editing. JN: Data curation, Methodology, Writing – original draft, Writing – review & editing. JO: Data curation, Investigation, Methodology, Validation, Writing – original draft, Writing – review & editing. FD: Validation, Writing – original draft, Writing – review & editing. JJ-L: Conceptualization, Project administration, Validation, Writing – original draft, Writing – review & editing. KD: Formal Analysis, Software, Supervision, Validation, Writing – original draft, Writing – review & editing.
